# Three‐dimensional analysis of shape variations and symmetry of the fibula, tibia, calcaneus and talus

**DOI:** 10.1111/joa.12900

**Published:** 2018-11-04

**Authors:** Nazlı Tümer, Vahid Arbabi, Willem Paul Gielis, Pim A. de Jong, Harrie Weinans, Gabrielle J. M. Tuijthof, Amir A. Zadpoor

**Affiliations:** ^1^ Department of Biomechanical Engineering Delft University of Technology (TU Delft) Delft The Netherlands; ^2^ Department of Orthopedics UMC Utrecht Utrecht The Netherlands; ^3^ Department of Mechanical Engineering Faculty of Engineering University of Birjand Birjand Iran; ^4^ Department of Radiology UMC Utrecht Utrecht The Netherlands; ^5^ Department of Orthopaedic Surgery Academic Medical Center University of Amsterdam Amsterdam Movement Sciences Amsterdam The Netherlands; ^6^ Research Centre Smart Devices Zuyd University of Applied Sciences Heerlen The Netherlands

**Keywords:** bilateral symmetry, calcaneus, fibula, subtalar joint, talocrural joint, talus, tibia

## Abstract

The bones forming the talocrural joint (TCJ) and subtalar joint (STJ) are often assumed to be bilaterally symmetric. Therefore, the contralateral limb (i.e. the fibula, tibia, calcaneus and talus) is used as a template or an intra‐subject control in clinical and research practice. However, the validity of the symmetry assumption is controversial, because insufficient information is available on the shape variations and bilateral (a)symmetry of the fibula, tibia, calcaneus and talus. Using three‐dimensional spatially dense sampled representations of bone shapes extracted from bilateral computed tomography scans of 66 individuals (55 male, mean age: 61 ± 10 years; 11 female, mean age: 53 ± 15 years), we analyzed whether: (i) similar shape patterns exist in the left and right bones of the same type; (ii) gender has an effect on bone shape variations; (iii) intra‐subject shape variation is smaller than that of inter‐subject for a given shape variance direction. For the first set of analyses, all left and right instances of the same type of bone were considered as two separate groups, and statistically compared with each other on multiple aspects including group location (central tendency), variance‐covariance scale (dispersion) and orientation (covariance structure) using distance‐based permutational tests. For the second and third sets of analyses, all left and right bones of the same type were pooled into one group, and shape variations in the TCJ and STJ bones were extracted using principal component analysis. The effects of gender on age‐adjusted bone shape differences were assessed using an analysis of covariance. Moreover, intra‐class correlation was employed to evaluate intra‐ and inter‐subject bone shape variations. For each bone type, both sides had similar shape patterns (*P*_permutational_‐values > 0.05). After Bonferroni adjustment, gender led to shape differences, which were mainly in the lateral and medial condyles of the tibia (*P *= 0.003), the length and height of the calcaneus (*P *< 0.001), the posterior and anterior talar articular surfaces of the calcaneus (*P *= 0.001), and in the posterior aspect of the talus (*P *= 0.001). Intra‐subject shape variations in the tibial tuberosity together with the diameter of the tibia, and the curvature of the fibula shaft and the diameter of the fibula were as high as those of inter‐subject. This result suggests that the shape symmetry assumption could be violated for some specific shape variations in the fibula and tibia.

## Introduction

There has been long‐standing interest in the geometric (Auerbach & Ruff, 2006; Dargel et al. 2009; Young et al. 2013; Radzi et al. 2014; Eckhoff et al. 2016) and non‐geometric (e.g. bone mineral density, structural stiffness, moment of areas; Pierre et al. 2010; Cristofolini et al. 2014) bilateral symmetry of the lower extremities. This interest is partly due to the symmetry assumption that is frequently made in clinical assessments and research studies. Some examples are summed. First, a common clinical practice is to use the contralateral unaffected side as a template for planning corrective osteotomy (Dobbe et al. 2011; Santoro et al. 2014). Second, the contralateral unaffected side is often used as a reference in arthroplasty surgeries to determine the size of an implant and its position, when the limb of interest is deformed by a fracture or a degenerative disease [e.g. osteoarthritis (OA); Pierre et al. 2010; Young et al. 2013; Ten Berg et al. 2016]. Third, the unaffected contralateral side usually serves as an intra‐subject control or as a shape template in research studies that assess whether a bone shape can be a risk factor for the onset of an injury (e.g. acute knee injury; Shultz & Nguyen, 2007; Smith et al. 2012) or a lesion (e.g. osteochondral defect) caused by an injury (e.g. lateral ankle sprain; Tümer et al. 2016).

For the symmetry assumption to be valid, it is necessary to establish that the differences in the geometric and non‐geometric features of the left and right extremities are sufficiently small. Nevertheless, limited information is available regarding the (a)symmetry of the lower extremities within and across populations. In particular, little is known about the shape variations and (a)symmetry of the bones forming the talocrural joint (TCJ) and subtalar joint (STJ) (i.e. the fibula, tibia, calcaneus and talus). The few studies that are available (Auerbach & Ruff, 2006; Daud et al. 2013; Radzi et al. 2014; Eckhoff et al. 2016) are limited due to small sample sizes, two‐dimensional (2D) data, or small number of points representing the three‐dimensional (3D) shape. Therefore, the (in)validity of the symmetry assumption for the fibula, tibia, calcaneus and talus is not yet established.

To gain insight in shape variations and (a)symmetry of the TCJ and STJ bones, we first analyzed whether similar shape patterns exist in left and right bones of the same type, and whether side bias (i.e. directional asymmetry; Palmer, 1994; Claes et al. 2012) appears. We then evaluated intra‐ and inter‐subject bone shape variations in principal directions, which expressed the most variance in the shapes of bone samples. Moreover, the effects of gender on age‐adjusted bone shape variations were assessed, as gender can lead to anatomical differences in lower extremities (Auerbach & Ruff, 2006; Bellemans et al. 2010; Unnanuntana et al. 2010; Daud et al. 2013; Lindner et al. 2013; Young et al. 2013; Wise et al. 2016). Unlike previous studies that have reduced the bone shape to a few anatomical landmarks (Auerbach & Ruff, 2006; Daud et al. 2013), we used 3D spatially dense descriptions of the TCJ and STJ bones together with advanced statistical techniques: a previously applied 3D statistical shape modeling method (van de Giessen et al. 2012; Tümer et al. 2016) and distance‐based permutational statistics (Claes et al. 2012, 2015).

## Materials and methods

**Figure 1:**
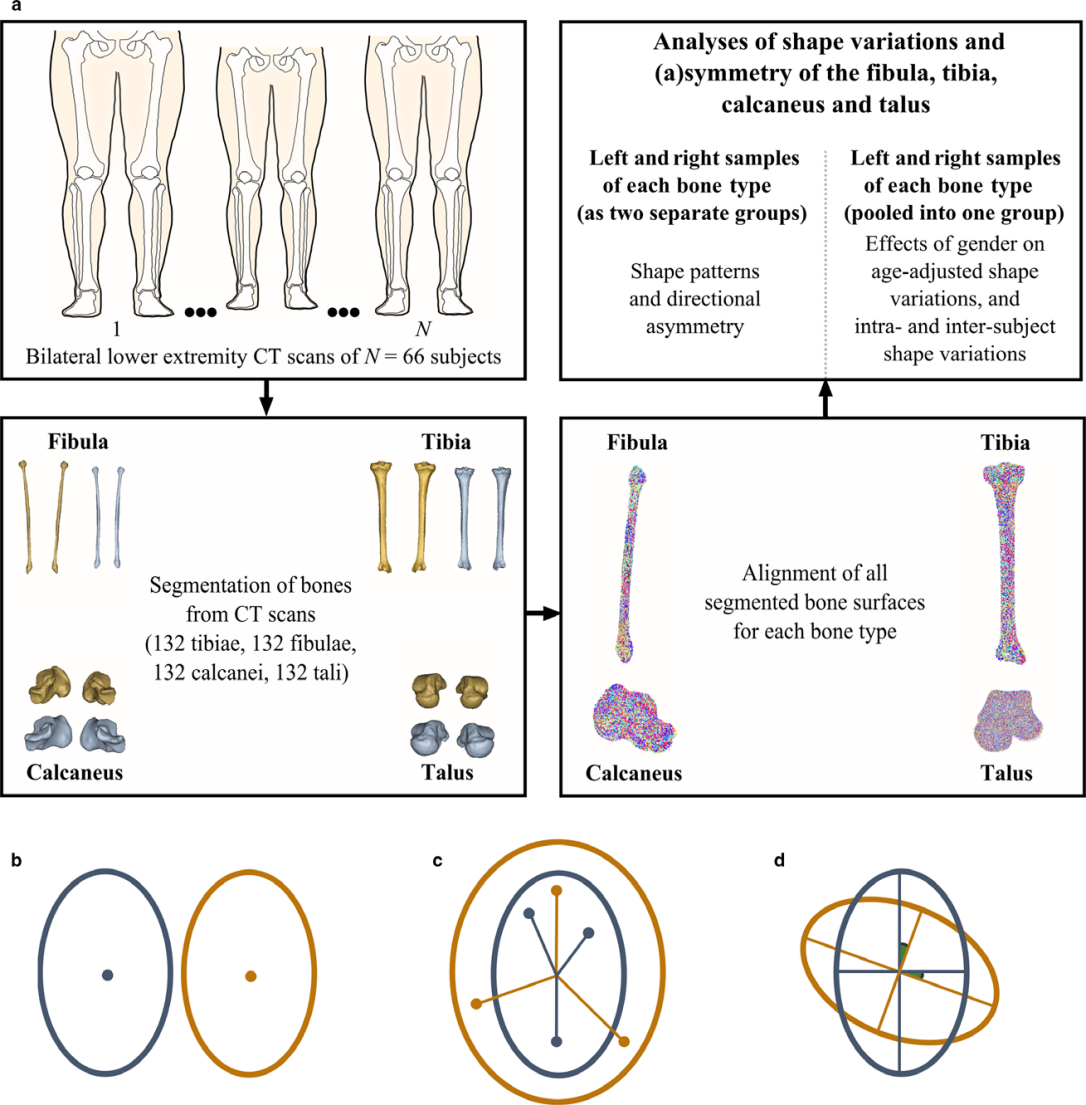
(a) A flow diagram of the study. Bilateral computed tomography (CT) scans were collected from 66 subjects. Left and right fibulae, tibiae, calcanei and tali were segmented from each CT scan. All bone samples of the same type were aligned into a common coordinate frame using an unbiased registration algorithm. For each bone type, shape variations and (a)symmetry were evaluated. (b–d) Multiple aspect analysis: group location, variance‐covariance scale and orientation. Two groups that show a difference in (b) their locations, (c) variance‐covariance scale and (d) variance‐covariance orientation only. In the group location, variance‐covariance scale and orientation tests, the features to concentrate on are (b) the sample mean, (c) the sample dispersion from the centroid, and (d) the sample subspace described using eigenvectors and the principal angles between them, respectively.

The flow‐chart presented in Fig. 1a outlines the main steps followed in this study. Briefly, in the first step, bilateral computed tomography (CT) scans of individuals were collected. In the second step, both left and right TCJ and STJ bones were segmented from all CT scans, and triangulated bone surfaces were extracted from the segmentation results. In the third step, all bone surfaces of the same type were aligned into a common coordinate frame in such a way that the differences due to position, orientation and scaling among bone instances were minimized. In the final step, the shape variations and (a)symmetry of the TCJ and STJ bones were analyzed. For each bone type, left and right bone samples were first considered as two separate groups and compared with each other on multiple aspects (i.e. group location, variance‐covariance scale and orientation) using permutational statistics. The group location test (Fig. 1b) was performed to assess side‐difference in the mean fibula (or tibia or calcaneus or talus) shape, in other words, the difference in central tendency. The variance‐covariance scale (Fig. 1c) and orientation (Fig. 1d) tests were employed to analyze side‐differences in the dispersion (i.e. the magnitude of shape variance only) and the shape variance directions around the mean fibula (or tibia or calcaneus or talus) shape, respectively. Following these analyses, all left and right bones of the same type were pooled into one group. Shape variations were extracted and statistically (i.e. an analysis of covariance, ancova) compared between females and males after adjusting them for the effects of age. Moreover, intra‐ and inter‐subject shape variations were assessed using the intraclass correlation coefficient (ICC). Each step is explained in detail in the following subsections.

### Data collection

Bilateral CT scans of patients who had undergone CT scanning due to unrelated medical reasons (i.e. vascular indications) were collected from the Utrecht Medical Center (UMC, Utrecht, The Netherlands). Each CT scan was evaluated by a trained medical doctor for signs of radiological OA in the TCJs. Any CT scan exhibiting signs of moderate or severe ankle OA (unilateral or bilateral; Cohen et al. 2015) was excluded from the study. The final dataset consisted of 66 CT scans collected from 66 out of 99 individuals. The scans were anonymized. Only the gender and age (55 male, mean age: 61 years, SD = 10 years; 11 female, mean age: 53 years, SD = 15 years) of the patients were available to us. For the use of CT scans in this study, an approval from the Medical Ethical Committee of UMC was not necessary.

All CT scans were acquired either using Philips Brilliance 64 or Philips iCT scanner (Philips Medical Systems, Best, The Netherlands). The acquisition parameters were: tube voltage 120 kVp, effective dose 150 mAs and slice thickness 1 mm. Tomographic reconstructions were made with a field of view covering both legs, a slice increment of 0.7 mm, and a matrix size of 512 × 512 pixels. The iDOSE4 reconstruction algorithm was used. Voxel sizes varied between 0.63 mm × 0.63 mm × 0.70 mm and 0.98 mm × 0.98 mm × 0.70 mm.

### Segmentation of bones from CT scans

All left and right bones of the same type (i.e. 132 fibulae, 132 tibiae, 132 calcanei and 132 tali) were segmented from the CT scans (Fig. 1a) using the freely available interactive graph‐cut segmentation software MITK‐GEM (Pauchard et al. 2016). The triangulated bone surfaces were extracted from the segmentations. All right bones were mirrored in the sagittal plane.

### Registration of bones

All bone surfaces of the same type were brought into a common coordinate frame (Fig. 1a) using an unbiased registration algorithm (van de Giessen et al. 2012; Tümer et al. 2016; Section 1 in Appendix S1), which enabled us to minimize differences due to position, orientation and scaling of bones. The registration parameters including the scale parameter for the mixture of Gaussians, *σ*, the number of points in the mean cloud, *n*_m_, and the trade‐off parameter, *λ* (van de Giessen et al. 2012) were retrieved from Tümer et al. (2016) for all tali (*σ* *= *3 mm, *n*_m_ = 2000, *λ* = 10^−6^). The registration parameters needed for alignment of the fibulae (*σ* *= *3 mm, *n*_m_ = 2000, *λ* = 10^−4^), tibiae (*σ* = 3 mm, *n*_m_ = 2000, *λ* = 10^−5^) and calcanei (*σ* *= *3 mm, *n*_m_ = 2000, *λ* = 5 × 10^−4^) were defined based on the outcomes of numerical experiments performed in a way described previously (van de Giessen et al. 2012; Tümer et al. 2016; Section 1 in Appendix S1).

### Statistical analyses ipsi‐ and contralateral sides as separate groups

Following the registration process, dense correspondence between aligned bone surfaces of the same type was automatically established (van de Giessen et al. 2009; Tümer et al. 2016; Section 2.1 in Appendix S1). The number of corresponding points settled on all aligned fibulae (*n *= 12 465), tibiae (*n *= 31 496), calcanei (*n *= 7717) and tali (*n *= 5541) was approximately 0.7 times the bone surface area averaged over all bone samples of the same type.

For the first set of comparisons, the left and right fibulae (or tibiae or calcanei or tali) were considered as two separate groups and compared with each other on multiple aspects, including group location, variance‐covariance scale and orientation using distance‐based permutational approaches in a similar manner as described in Claes et al. (2012, 2015). The Euclidean distance between the means of two groups was employed as *D*(*istance*)‐statistic in the group location test (Claes et al. 2012, 2015; Section 2.2 in Appendix S1). The left and right fibulae (or tibiae or calcanei or tali) were permutated 10 000 times (*N*_perm_) across groups, and the *D*‐statistic was calculated at each permutation (*D*‐statistic_perm_). A *P*‐value assessed under permutation (*P*_perm_) was determined by:

(1) $$P_{\mathrm{perm}}=\frac{N_{i}}{N_{\mathrm{perm}}}$$

where *N*_*i*_ represents the number of cases in which permutated values are higher or equal to *D*‐statistic (i.e. *D*‐statistic_perm _≥ *D*‐statistic).

The *D*‐statistic used in the variance‐covariance scale test was the absolute difference in the average residual of the two groups (Claes et al. 2012, 2015; Section 2.3 in Appendix S1). The permutations were realized, and permutated values (i.e. *D*‐statistic_perm_) were obtained in exactly the same way as described for the group location test. A *P*_perm_ *‐*value was calculated using Eq. (1).

**Figure 2:**
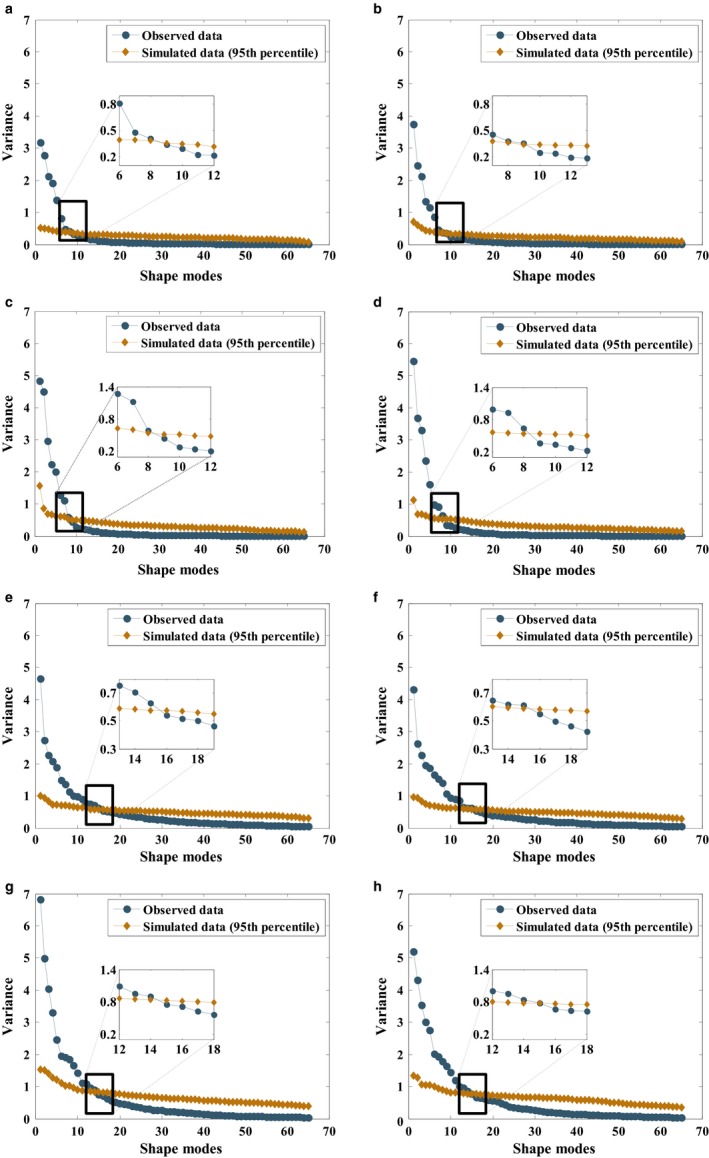
The scree plots with parallel analyses (PAs) are given for (a) left fibulae, (b) right fibulae, (c) left tibiae, (d) right tibiae, (e) left calcanei, (f) right calcanei, (g) left tali and (h) right tali. Blue and dark gold markers stand for observed and simulated data, respectively. All the principal components (PCs) up to the one found at the intersection of two lines (lines represented with blue and dark gold colors) were retained and used in the variance‐covariance orientation test and description of bone shape variations within a studied population. Accordingly, the number of PCs kept is (a, b) 8 for left and right fibulae, (c, d) 8 for left and right tibiae, (e, f) 15 for left and right calcanei, and (g, h) 14 for left and right tali.

In the variance‐covariance orientation test, two shape subspaces represented with eigenvectors [i.e. principal components (PCs) or modes of shape variation] and the principal angles between them were compared. To obtain the shape subspace of the left bones of the same type, a principal component analysis (PCA) was performed on the covariance matrix of the data vectors that consisted of the 3D coordinates of the corresponding points established on all left bones. The shape subspace of the right bones of the same type was obtained in the same manner. The *D*‐statistic used in the orientation test was the projection metric (Hamm & Lee, 2008; Claes et al. 2012, 2015):

(2) $$D_{k}=\sqrt{k-\sum_{i=1}^{k}\cos^{2}\theta_{i}}$$

in which *θ*_*i*_ {*i *= 1, …, *k*} are the principal angles (Knyazev & Argentati, 2002; Taylor & Krzanowski, 2012) and *k* is the number of PCs to be kept. To determine the number of PCs (*k*) to retain, a parallel analysis (PA; Ledesma et al. 2007; Section 2.4 in Appendix S1) was carried out (Fig. 2). A set of *k* distances (*D*_k_, *D*‐statistic) was calculated based on Eq. (2) by incrementally increasing the number of principal angles from 1 to *k*. Then, the left and right bones of the same type were permutated 10 000 times across groups. At each permutation, the shape subspaces of the left and right bones of the same type were re‐determined using the permutated data, and a set of *k* distances (*D*_*k*_, *D*‐statistic_perm_) was computed. All *P*_perm_ values were determined based on Eq. (1).

The group location, variance‐covariance scale and orientation tests were carried out using the statistical routines developed (Claes et al. 2012) in Matlab (Matlab R20013b, The Mathworks, Natick, MA, USA).

### Statistical analyses ipsi‐ and contralateral sides pooled into one group

To describe the shape variations in the bones of the same type and analyze the side‐shape differences in given shape variance directions, ipsi‐ and contralateral bones of the same type were pooled into one group and PCA was applied on the covariance matrix of the combined data vectors (Tümer et al. 2016). As a result, the shape parameters (Section 3.1 in Appendix S1) were obtained for each bone type, which expressed how much each bone sample deviated from the mean bone shape in given shape variance directions (i.e. PCs, eigenvectors, or modes of shape variation; Sarkalkan et al. 2014; Tümer et al. 2016).

A Kolmogorov–Smirnoff test was carried out to evaluate whether the shape parameters given for each of the *k* PCs conformed to a normal distribution. The shape variations in each bone type adjusted for the effects of age were compared between males and females using an ancova. Moreover, the intra‐subject shape variation was compared with that of inter‐subject for each of the *k* PCs using the ICC. A single measurement, absolute‐agreement, and two‐way random effects model (Koo & Li, 2016) was employed for the latter analyses. All ICC estimates and their 95% confidence intervals (CIs) were reported. An ICC estimate of 1 indicated perfect symmetry within an individual. In other words, the total bone shape variation was described by the inter‐subject shape difference only. In contrast, an ICC estimate of 0 implied that the intra‐ and inter‐subject shape variations were equal to each other. When the 95% CI of the ICC for a PC included zero, we deemed the PC not significantly symmetrical. All the statistical analyses were conducted using SPSS (Version 22, Chicago, IL, USA).

## Results

### Shape patterns in ipsi‐ and contralateral sides as separate groups

The *P*_perm_ values were higher than the statistical significance level of 0.05 for the group location, variance‐covariance scale and orientation tests (Table 1). Therefore, the side‐differences in the mean shape of the fibula, tibia, calcaneus and talus were not statistically significant (Table 1, group location test). Moreover, variations (Table 1, variance‐covariance scale) and differences in shape variance directions (Table 1, variance‐covariance orientation) around the mean shape of each bone type were not significantly different between left and right.

**Table 1:** *D*_stat_ and *P*_perm_ values resulting from the group location (1st row), variance‐covariance scale (2nd row) and orientation (3rd–17th rows) tests performed for the fibula, tibia, calcaneus and talus.

	Fibula		Tibia		Calcaneus		Talus	
	*D*_stat_	*P*_perm_	*D*_stat_	*P*_perm_	*D*_stat_	*P*_perm_	*D*_stat_	*P*_perm_
Location Scale Orientation 1 : 1 : 15 principal angles	0.53	0.84	0.68	0.74	0.62	1.00	0.87	0.98
	0.08	0.60	0.00	0.99	0.04	0.77	0.16	0.42
	0.13	0.98	0.11	1.00	0.20	1.00	0.22	0.92
	0.20	0.99	0.16	1.00	0.35	1.00	0.35	0.97
	0.28	0.99	0.24	1.00	0.47	1.00	0.44	1.00
	0.37	0.95	0.30	1.00	0.58	1.00	0.57	0.99
	0.46	0.98	0.37	1.00	0.68	1.00	0.69	0.98
	0.57	0.99	0.46	1.00	0.79	1.00	0.80	1.00
	0.77	0.99	0.56	1.00	0.91	1.00	0.92	1.00
	1.25	0.94	0.73	1.00	1.02	1.00	1.04	1.00
	–	–	–	–	1.15	1.00	1.17	1.00
	–	–	–	–	1.29	1.00	1.34	1.00
	–	–	–	–	1.44	1.00	1.51	1.00
	–	–	–	–	1.60	1.00	1.70	1.00
	–	–	–	–	1.77	1.00	1.95	1.00
	–	–	–	–	2.01	1.00	2.19	1.00
	–	–	–	–	2.23	1.00	–	–

*D*_stat_ represents the Euclidean distance between the means of left and right groups (the group location test), the absolute difference in the average residual of the left and right groups (the variance‐covariance scale test), and the projection metric (the variance‐covariance orientation test). *P*_perm_ describes a *P*‐value obtained under *N*_perm_ = 10 000 permutations.

### Shape variations in ipsi‐ and contralateral sides pooled into one group

Principal components kept for each bone type (Fig. 2; i.e. *k *= 8 for the fibula and tibia, *k *= 15 for the calcaneus, and *k *= 14 for the talus) were used to describe shape variations. These PCs explained 79, 84, 67 and 72% of the total shape variation in the fibula, tibia, calcaneus and talus, respectively.

None of the *P*‐values resulting from a Kolmogorov–Smirnoff test was lower than the statistical significance level of 0.05, meaning that the shape parameters of all 132 fibulae (or tibiae or calcanei or tali) given for a specific PC came from a normal distribution.

**Figure 3:**
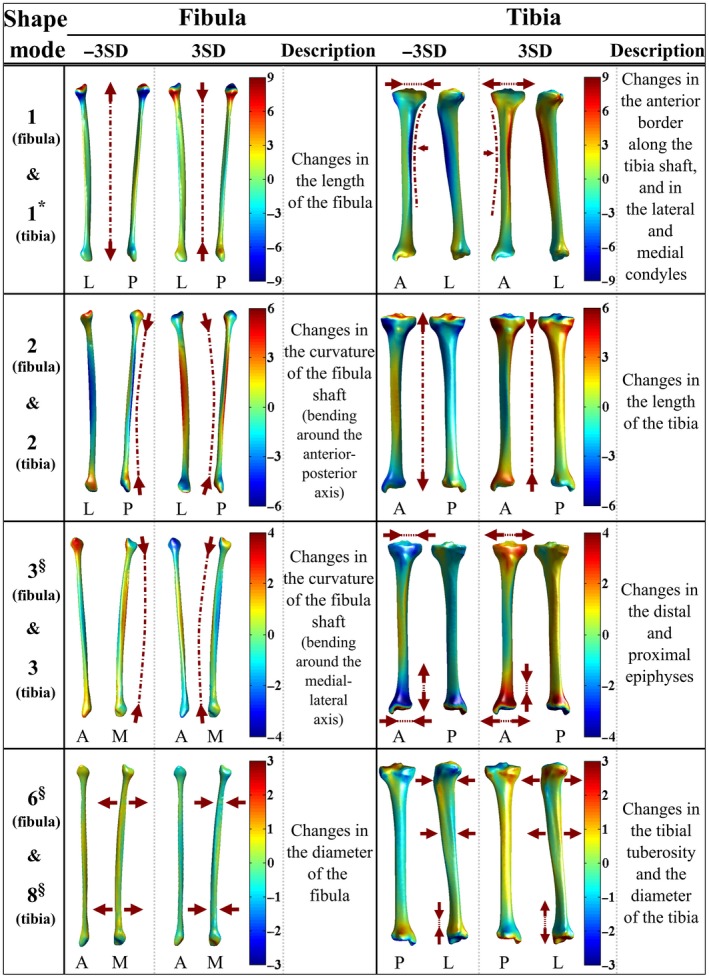
The first three rows display deviations (mm) of the fibula and tibia from the mean fibula shape (left column) and the mean tibia shape (right column) in the positive (+3 SD) and negative (−3 SD) directions of the first three principal components (PCs) of the fibula and tibia, respectively. Shape variations of the fibula and tibia explained by PC 6 of the fibula and PC 8 of the tibia, respectively, are shown in the fourth row. The shape variance directions that expressed significantly different shape variations between females and males are marked with ‘*’. The marker ‘§’ is used to indicate shape variance directions for which intra‐ and inter‐subject shape variations were comparable to each other.
- (PC 2 of the fibula; Fig. 3) the curvature of the fibula shaft (bending around the anterior–posterior axis);
- (PC 3 of the fibula; Fig. 3) the curvature of the fibula shaft (bending around the medial–lateral axis);
- (PC 1 of the tibia; Fig. 3) the anterior border along the tibia shaft, and in the lateral and medial condyles;
- (PC 2 of the tibia; Fig. 3) the length of the tibia;
- (PC 3 of the tibia; Fig. 3) the distal and proximal epiphyses;
- (PC 1 of the talus; Fig. 4) the lateral rotation of the talar head;

For each bone type, changes observed along the first three PCs were in:

- (PC 1 of the fibula; Fig. 3) the length of the fibula;

**Figure 4:**
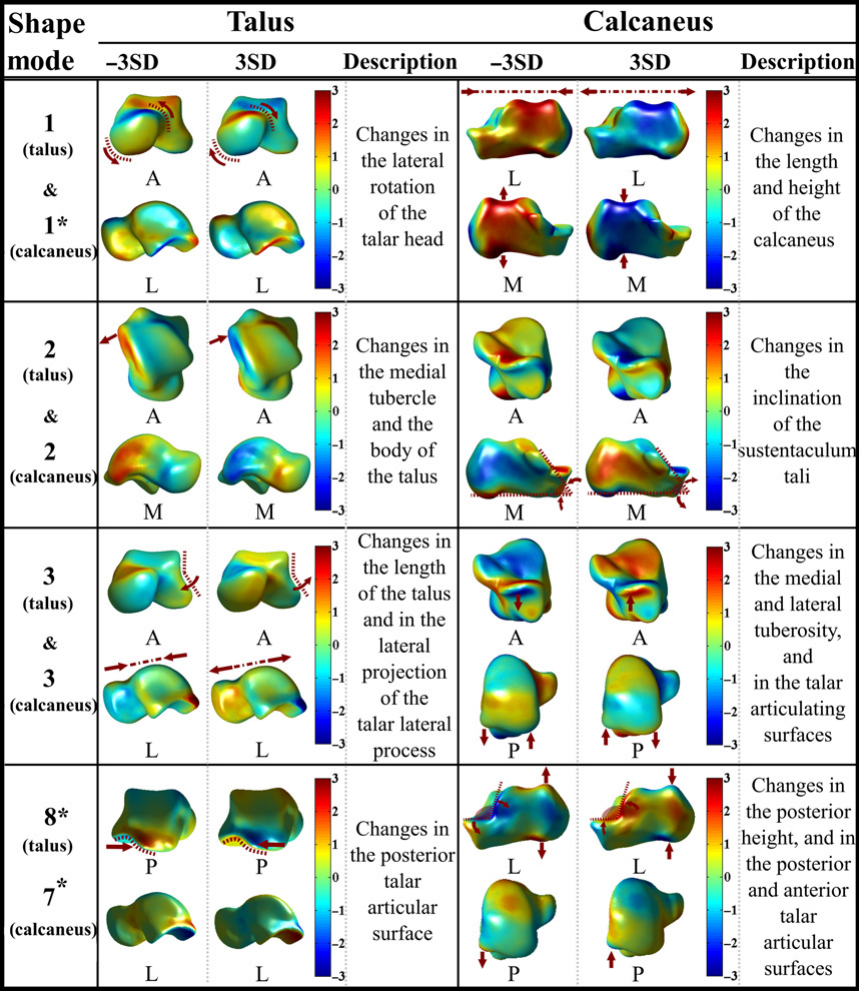
The first three rows display deviations (mm) of the talus and calcaneus from the mean talus shape (left column) and the mean calcaneus shape (right column) in the positive (+3 SD) and negative (−3 SD) directions of the first three principal components (PCs) of the talus and calcaneus, respectively. Shape variations in the talus and calcaneus explained by PC 8 of the talus and PC 7 of the calcaneus are shown in the fourth row. Shape variance directions that expressed significantly different shape variations between females and males are marked with ‘*’.
- (PC 2 of the talus; Fig. 4) the medial tubercle and the body of the talus;
- (PC 3 of the talus; Fig. 4) the length of the talus, and in the lateral projection of the talar lateral process;
- (PC 1 of the calcaneus; Fig. 4) the length (i.e. anterior–posterior direction) and height (i.e. superior–inferior direction) of the calcaneus;
- (PC 2 of the calcaneus; Fig. 4) the inclination of the sustentaculum tali;
- (PC 3 of the calcaneus; Fig. 4) the medial and lateral tuberosity, and in the talar articulating surfaces.

Shape changes described by other remaining PCs are not presented here. The reasoning behind the choice made is that PCs higher than PC 3 explained relatively small shape variations distributed over the bone surfaces and most of them, except a few mentioned in the following two subsections (see ‘Effects of gender on age‐adjusted shape variations’ and ‘Intra‐ and inter‐subject shape variations’ sections), did not express significantly different bone shape variations between males and females, nor did they describe higher intra‐subject shape variations than inter‐subject.

#### Effects of gender on age‐adjusted shape variations

After adjusting the statistical significance level of 0.05 with Bonferroni, one PC for the tibia (i.e. PC 1 of the tibia, *P *= 0.003), one PC for the talus (i.e. PC 8 of the talus, *P *= 0.001) and two PCs for the calcaneus (i.e. PC 1, *P *< 0.001 and PC 7 of the calcaneus, *P *= 0.001) expressed significant shape differences between the tibiae, tali and calcanei of male and female subjects, respectively (Table 2). Shape changes observed along PC 1 of the tibia (Fig. 3) and PC 1 of the calcaneus (Fig. 4) are presented in the previous section. Regarding PC 7 of the calcaneus (Fig. 4), shape changes were mainly in the posterior height, and in the posterior and anterior talar articular surfaces. PC 8 of the talus (Fig. 4) expressed changes in the posterior aspect of the talus. The shape variations in the fibula, tibia, calcaneus and talus described by the other remaining PCs did not significantly differ based on gender (Table 2).

**Table 2:** *P*‐values resulting from ancova tests

Shape mode	Fibula	Tibia	Calcaneus	Talus
1	0.341	0.003*	0.000*	0.219
2	0.406	0.777	0.589	0.426
3	0.735	0.971	0.159	0.476
4	0.032	0.045	0.013	0.521
5	0.056	0.278	0.005	0.043
6	0.901	0.844	0.022	0.248
7	0.047	0.583	0.001*	0.657
8	0.086	0.061	0.068	0.001*
9	–	–	0.236	0.703
10	–	–	0.273	0.036
11	–	–	0.661	0.922
12	–	–	0.336	0.135
13	–	–	0.911	0.024
14	–	–	0.143	0.957
15	–	–	0.334	–

The *P*‐values under Bonferroni adjusted significance level of 0.006 (= 0.05/8) for the fibula and tibia, 0.003 (= 0.05/15) for the calcaneus and 0.004 (= 0.05/14) for the talus are marked with ‘*’.

**Figure 5:**
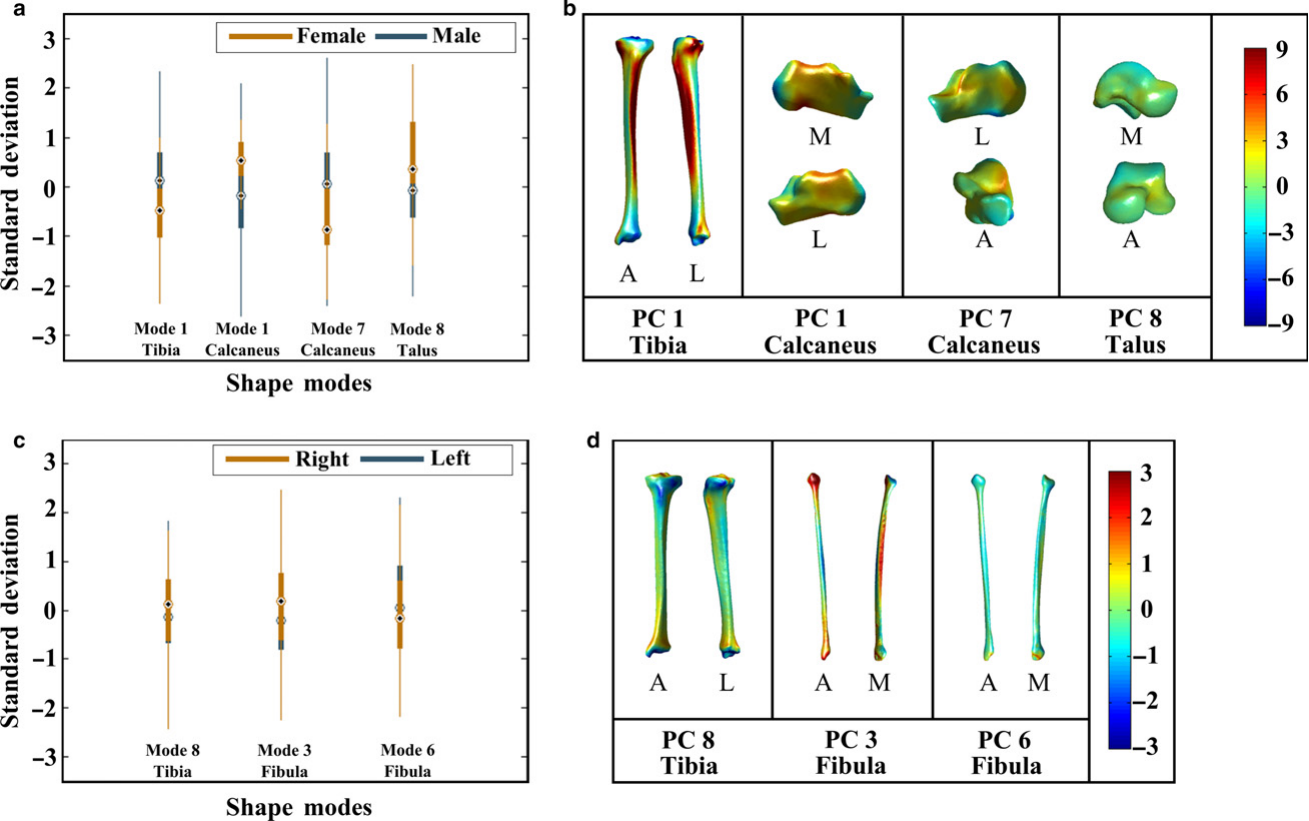
(a) Box plots showing the distributions of shape parameters observed along principal component (PC) 1 of the tibia, PC 1 and PC 7 of the calcaneus, and PC 8 of the talus. These PCs described statistically significant shape variations between males (blue color) and females (dark gold color). (b) Point‐to‐surface distances (mm) calculated between female and male (i) tibiae deviating from each other the most along PC 1 of the tibia, (ii) calcanei deviating from each other the most along PC 1 of the calcaneus, (iii) PC 7 of the calcaneus, and (iv) tali deviating from each other the most along PC 8 of the talus. (c) The distributions of shape parameters observed for the left (blue color) and right (dark gold color) sides along PC 8 of the tibia, PC 3 and PC 6 of the fibula. Intra‐subject shape variations in the tibia and fibula described by these PCs were similar to those of inter‐subject. (d) Point‐to‐surface distances (mm) calculated between left and right (i) tibiae deviating from each other the most along PC 8 of the tibia, (ii) fibulae deviating from each other the most along PC 3 of the fibula, and (iii) PC 6 of the fibula.

The box plots showing the distribution of shape parameters (Section 3.1 in Appendix S1) observed within the studied population along PC 1 of the tibia, PC 1 and PC 7 of the calcaneus, and PC 8 of the talus are provided in Fig. 5a. The means of the shape parameters (i.e. along PC 1 of the tibia) for the tibiae of females and males were −0.568 and 0.114, respectively. Accordingly, females had relatively smaller lateral and medial condyles (deviation from the mean tibia shape in the negative direction of the PC 1 of the tibia; Fig. 3) on average as compared with those of males (deviation from the mean tibia shape in the positive direction of the PC 1 of the tibia; Fig. 3). The means of the shape parameters for the calcanei of females along PC 1 and PC 7 of the calcaneus were 0.678 and −0.627, respectively (Fig. 5a). With reference to the calcanei of males, the means of the shape parameters were −0.1355 and 0.1253 along PC 1 and PC 7 of the calcaneus, respectively (Fig. 5a). Deviation from the mean calcaneus shape in the negative direction of the PC 1 of the calcaneus (Fig. 4) expressed the shortening and enlargement of the calcaneus in length (i.e. anterior–posterior direction) and height (i.e. superior–inferior direction), respectively. Observed changes in the calcanei having positive shape parameters along PC 1 of the calcaneus (Fig. 4) were vice versa. Therefore, calcanei of females were on average longer in length (i.e. anterior–posterior direction) and shorter in height (i.e. superior–inferior direction) as compared with those of males. Deviation from the mean calcaneus shape in the negative direction of the PC 7 of the calcaneus, as observed in the calcanei of females (i.e. the mean of the shape parameters = −0.627), expressed the enlargement of an angle located between the posterior and anterior talar articular surfaces, and the lateral process of calcaneal tuberosity. The means of the shape parameters observed along PC 8 of the talus were 0.492 and −0.098 for females and males, respectively (Fig. 5a). Changes observed along PC 8 of the talus (Fig. 4) were in the posterior aspect of the talus. Deviation from the mean talus shape in the negative direction of this PC displayed enlargement of the posterior talar articular contour (Fig. 4), suggesting that tali of males had larger posterior aspect on average.

Point‐to‐surface distance is presented in Fig. 5b, which was calculated between the female and male tibiae deviating from each other the most along PC 1 of the tibia. Similarly, three other point‐to‐surface distances calculated for the cases: (i) PC 1; and (ii) PC 7 of the calcaneus; and (iii) PC 8 of the talus, are given in Fig. 5b. Referring to Fig. 5b, point‐to‐surface distances exceeding 9 mm, 4 mm and 2 mm were observed between the tibiae (i.e. varied along PC 1 of the tibia), calcanei (i.e. varied along PC 1 or PC 7 of the calcaneus) and tali (i.e. varied along PC 8 of the talus) of females and males, respectively.

#### Intra‐ and inter‐subject shape variations

For all of the 45 PCs, except two for the fibula (i.e. PC 3 and PC 6 of the fibula) and one for the tibia (i.e. PC 8 of the tibia), intra‐subject shape variations were smaller than inter‐subject variations (Table 3). The ICC estimates and their 95% CI (Table 3) were 0.03 (−0.21 to 0.27), −0.04 (−0.28 to 0.20) and 0.06 (−0.19 to 0.29) for the PC 3 and PC 6 of the fibula, and PC 8 of the tibia, respectively. These PCs described changes in the curvature of the fibula shaft and the diameter of the fibula (Fig. 3 PC 3 and PC 6 of the fibula), and in the tibial tuberosity together with the diameter of the tibia (Fig. 3 PC 8 of the tibia).

**Table 3:** ICC estimates and their 95% CIs for the fibula, tibia, calcaneus and talus.

Shape mode	Fibula		Tibia		Calcaneus		Talus	
	ICC	95% CI	ICC	95% CI	ICC	95% CI	ICC	95% CI
1	0.72	0.58–0.82	0.85	0.77–0.91	0.95	0.92–0.97	0.56	0.37–0.71
2	0.74	0.60–0.83	0.50	0.29–0.66	0.84	0.75–0.90	0.35	0.12–0.54
3	0.03*	−0.21 to 0.27	0.48	0.27–0.64	0.85	0.77–0.91	0.55	0.35–0.69
4	0.47	0.25–0.64	0.43	0.22–0.61	0.82	0.72–0.88	0.43	0.22–0.61
5	0.67	0.51–0.78	0.72	0.58–0.82	0.85	0.76–0.90	0.40	0.18–0.58
6	−0.04*	−0.28 to 0.20	0.87	0.80–0.92	0.72	0.58–0.82	0.27	0.03–0.48
7	0.66	0.49–0.77	0.59	0.41–0.73	0.90	0.85–0.94	0.67	0.51–0.78
8	0.40	0.18–0.58	0.06*	−0.19 to 0.29	0.74	0.61–0.83	0.67	0.51–0.78
9	–	–	–	–	0.80	0.70–0.88	0.45	0.23–0.62
10	–	–	–	–	0.82	0.72–0.88	0.59	0.41–0.73
11	–	–	–	–	0.83	0.74–0.90	0.49	0.28–0.65
12	–	–	–	–	0.82	0.73–0.89	0.29	0.06–0.50
13	–	–	–	–	0.81	0.70–0.88	0.37	0.14–0.56
14	–	–	–	–	0.80	0.69–0.87	0.46	0.25–0.63
15	–	–	–	–	0.67	0.51–0.79	–	–

Shape variance directions in which intra‐subject shape variations were comparable to those of inter‐subject are marked with ‘*’.

CI, confidence interval; ICC, independent component analysis.

The distributions of shape parameters observed along PC 8 of the tibia, and PC 3 and PC 6 of the fibula for both left and right sides are presented in Fig. 5c. The means of the shape parameters for left and right tibiae (i.e. varied along PC 8 of the tibia; Fig. 5c) were −0.028 and 0.028, respectively. Regarding the left fibulae, the means of the shape parameters observed along PC 3 of the fibula and PC 6 of the fibula (Fig. 5c) were −0.082 and 0.098, respectively. Similarly, the means of the shape parameters were 0.082 (i.e. along PC 3 of the fibula; Fig. 5c) and −0.098 (i.e. along PC 6 of the fibula; Fig. 5c) for the right fibulae.

Point‐to‐surface distance calculated between a pair of tibia is presented in Fig. 5d, which deviated from each other the most along PC 8 of the tibia. In the same way, point‐to surface distances are provided in Fig. 5d for two pairs of fibulae varying the most along PC 3 and PC 6 of the fibula. Referring to Fig. 5d, distances exceeding 2 mm were observed along the surfaces of each pair.

## Discussion

The bilateral symmetry of the TCJ and STJ bones is often assumed in clinical practice and research studies. Nevertheless, the validity of the symmetry assumption is not yet established due to limited information on the shape variations and (a)symmetry of the fibula, tibia, calcaneus and talus. In this study, using detailed 3D bone shape data and advanced statistical techniques, we addressed whether: (i) both sides of each bone type exhibit similar shape patterns, and a side bias (i.e. directional asymmetry; Palmer, 1994; Claes et al. 2012) exists; (ii) gender has an influence on bone shape differences; and (iii) intra‐subject shape variations are smaller than those of inter‐subject for given shape variance directions.

The ipsi‐ and contralateral sides of the TCJ and STJ bones (Table 1) had similar shape patterns. There was no indication for left or right bias in any bone type (Table 1). Behavioral studies on the lower limb laterality (Gentry & Gabbard, 1995; Bell & Gabbard, 2000) have found right‐footedness to be more prevalent. Nevertheless, it has also been stated that contralateral non‐preferred foot supports the activities (e.g. kicking, stamping) of the dominant foot by contributing to postural stability (Gentry & Gabbard, 1995; Bell & Gabbard, 2000; Auerbach & Ruff, 2006). Based on these studies, it seems plausible that contralateral non‐preferred extremity is subjected to more or less the same mechanical loads as the dominant limb. Therefore, left‐side or right‐side bias may not be present for the fibula, tibia, calcaneus and talus.

Gender led to tibial, calcaneal and talar shape differences in four shape variance directions (PC 1 of the tibia, Fig. 3; PC 1 and PC 7 of the calcaneus, Fig. 4; PC 8 of the talus, Fig. 4). Considering PC 1 of the tibia (Figs 3 and 5a), females had relatively smaller lateral and medial condyles. Our findings are in agreement with the outcomes of previous studies (Bellemans et al. 2010; Wise et al. 2016). In Bellemans et al. (2010), large variations in mediolateral dimensions were observed. Moreover, the smallest tibiae were predominantly found in females (Bellemans et al. 2010). In Wise et al. (2016), smaller tibial head widths were reported for females as compared with those of males. Regarding PC 8 of the talus (Figs 4 and 5a), tali of males had a relatively larger posterior aspect. This outcome is in line with previous studies (Harris & Case, 2012; Lee et al. 2015) that analysed the morphology of the talus and its sexual dimorphism. Furthermore, relatively larger values for the talar breadth and surface area have been reported for male tali (Harris & Case, 2012; Lee et al. 2015). On average, female calcanei in our study seem to be longer in length (i.e. anterior–posterior direction) and shorter in height (i.e. superior–inferior direction). This observation is not in full agreement with other studies (Riepert et al. 1996; Gualdi‐Russo, 2007; Harris & Case, 2012), as the average length and height of the calcaneus have been reported to be larger in males.

In this study, the sample size and the number of females involved in the dataset is limited. The disproportion of males and females could affect the generalizability of all the analyses, except the one performed to assess the effects of gender on age‐adjusted shape variations. In the latter analysis, the small dataset could have an effect on the statistical power. To analyze the effects of gender on age‐adjusted shape variations, multiple comparisons were performed. Therefore, the statistical significance level of 0.05 was adjusted according to Bonferroni with the aim of reducing type I errors. The cost of this correction is an increased probability of type II errors (i.e. reduced power). Another limitation of this study is that bone shape differences between females and males, and bilateral (a)symmetry could not be studied based on different age groups. The inclusion of younger and older individuals might impact the generalizability of the findings reported in this study. Future studies should aim for a larger and more representable population sample (e.g. more females), to increase statistical power and generalizability of the results.

Intra‐subject shape variations in the talus and calcaneus along each of *k* PCs (14 and 15 shape variance directions for the talus and calcaneus, respectively) were smaller than those of inter‐subject (Table 3). These results suggest that the shapes of the calcaneus and talus were more symmetric within an individual than between subjects. Due to a scarcity of information on bilateral shape (a)symmetry of the talus and calcaneus, it is not easy to compare the findings of this study with those of others. The study presented by Islam et al. (2014) is one of the references that can be referred to. Although the methods followed in Islam et al. (2014) are different than those presented here, their observations made using CT data of 11 intact tali (eight male and three female subjects) imply that the shape of talus is bilaterally symmetric. Regarding the calcaneus, intra‐subject variations within the anatomy were reported to be smaller than those of inter‐subject in Stephan et al. (2014) for the area and 3D orientation of the joint surfaces of the calcaneus. The outcomes of this study on the bilateral (a)symmetry of the talus and calcaneus, and those of the studies (Islam et al. 2014; Stephan et al. 2014) imply that the shapes of the talus and calcaneus are bilaterally symmetric, and the shape of the contralateral side can be used as a control during a surgery (e.g. anatomical reconstruction of the calcaneus of a patient with a calcaneus fracture) or as a shape template for implant design.

Intra‐subject shape variations were in general smaller than those of inter‐subject for the tibia and fibula (Table 3 95% CIs did not include zero for 13 out of 16 shape variance directions). Nevertheless, the curvature of the fibula shaft (bending around the medial–lateral axis, PC 3 of the fibula; Fig. 3), the diameter of the fibula (PC 6 of the fibula; Fig. 3) and the tibial tuberosity together with the diameter of the tibia (PC 8 of the tibia; Fig. 3) varied within a subject as much as between individuals (Table 3). One of the explanations for observing different (a)symmetry level in the cross‐sectional dimensions of the fibula and tibia as compared with those seen in other bone features, such as the length (i.e. PC 1 of the fibula and PC 2 of the tibia; Fig. 3) may be that different structural features within the same bone exhibit independent development. For example, the subperiostal growth of bone cortices could endure throughout life, although a long bone stops growing in length after the closure of the epiphyseal growth plate (Auerbach & Ruff, 2006). The cross‐sectional dimensions of skeletally mature weight‐bearing bones could be more sensitive to mechanical loadings. As bone cortices can grow, changes in the cross‐sectional dimensions could be observed while bone adapts to its mechanical environment. Referring to Ruff et al. (2006), the threshold at which bone deposition/reposition is stimulated is not constant, but varies with respect to several intrinsic (e.g. genetic factors, and the age and hormonal status of individuals) and extrinsic (e.g. loading history, the frequency of loading) factors. Considering these aspects, it seems plausible that bilateral differences could exist in bone stimulation threshold, bone deposition/reposition within individuals depending on their foot preference, and physical activities that can affect the contribution of none‐preferred limb to their postural stability. The cross‐sectional dimensions of the fibula and tibia may influence the determination of implant size and its placement in arthroplasty surgery, while the curvature of the fibula can be relevant for planning corrective osteotomy. Therefore, side‐shape differences in the fibula (PC 3 and PC 6 of the fibula; Fig. 3) and tibia (PC 8 of the tibia; Fig. 3) may adversely affect the success of an arthroplasty surgery and corrective osteotomy performed under shape symmetry assumption.

A strength of this study is that all bone samples were spatially dense sampled in 3D. This enabled us to cover bony regions that cannot be described with a set of conventional 2D or 3D measurements. Using 3D spatially dense data, we analyzed for the first time shape variations and (a)symmetry of all the bones forming the TCJ and STJ. Considering the nature of the PCA to describe shape variations, it is wise to mention that isolated locations of asymmetry may not be sufficiently captured (van de Giessen et al. 2011). An independent component analysis (ICA) could be used as an alternative to PCA to describe shape variations in a more localized way. We refer interested readers to Hyvärinen & Oja (2000) and Hyvärinen (2013) for the details on ICA. Although ICA has potential extracting substantially distinct features, it has been less often used in the area of statistical shape analysis (Wu et al. 2014). Therefore, PCA was preferred in this study, which is the most commonly used technique to describe shape variations (Wu et al. 2014; Zhao et al. 2015).

## Conclusions

We observed that both sides of the bones forming the TCJ and STJ exhibited similar shape patterns, and directional asymmetry did not exist in any bone type (i.e. fibula, tibia, calcaneus and talus). Gender did not explain, in general, significantly different shape variations in all the above‐mentioned bones. Nevertheless, four shape variance directions described statistically significant shape differences between the tibiae (i.e. changes in the anterior border along the tibia shaft, and in the lateral and medial condyles), calcanei (i.e. changes in the length and height of the calcaneus, and in the posterior height, and in the posterior and anterior talar articular surfaces) and tali (i.e. changes in the posterior aspect of the talus) of females and males, after Bonferroni adjustment. The shape symmetry assumption was in general valid. However, intra‐subject shape variations were as high as those of inter‐subject in the shape variance directions describing changes in the curvature of the fibula shaft, the diameter of the fibula, and the tibial tuberosity together with the diameter of the tibia. These observations indicate that the symmetry assumption may be violated. Deviation from symmetry in the fibula and tibia may adversely affect the outcomes of studies using the contralateral side as a shape template or intra‐subject control, and the success of an arthroplasty surgery or corrective osteotomy performed with shape symmetry assumption.

## Supporting information


**Appendix S1.** Technical Details for the Analysis of Shape Variations and Symmetry.
