# Supplementary material for: Three‐dimensional analysis of shape variations and symmetry of the fibula, tibia, calcaneus and talus
Source: J Anat. 2018 Nov 4;234(1):132–44. doi: 10.1111/joa.12900 (PMC6284442; doi:10.1111/joa.12900)
Supplement: Supplementary file 1 — Appendix S1. Technical Details for the Analysis of Shape Variations and Symmetry. [file JOA-234-132-s001.docx]

**SUPPLEMENTARY MATERIAL:**

1. **Registration of point clouds (i.e. bones represented with points)**

The unbiased registration algorithm (van de Giessen et al., 2012) can be used to register a large number of shapes (i.e. point clouds) (Tümer et al., 2016). The main idea behind the registration algorithm is to fit an evolving mean shape to each of the point cloud and to align the latter to the mean point cloud in the meantime. In this document, some of the important points are presented related to the unbiased registration algorithm. For detailed information, readers are referred to the study presented by van de Giessen (van de Giessen et al., 2012).

Figure S1 describes how correspondence between *N* (i.e. 66) number of point clouds *C_i_* (*i* = 1,…, *N*) and the evolving mean cloud *M* with *n*_m_ points (i.e. 2000) (Tümer et al., 2016) is established using the registration algorithm.


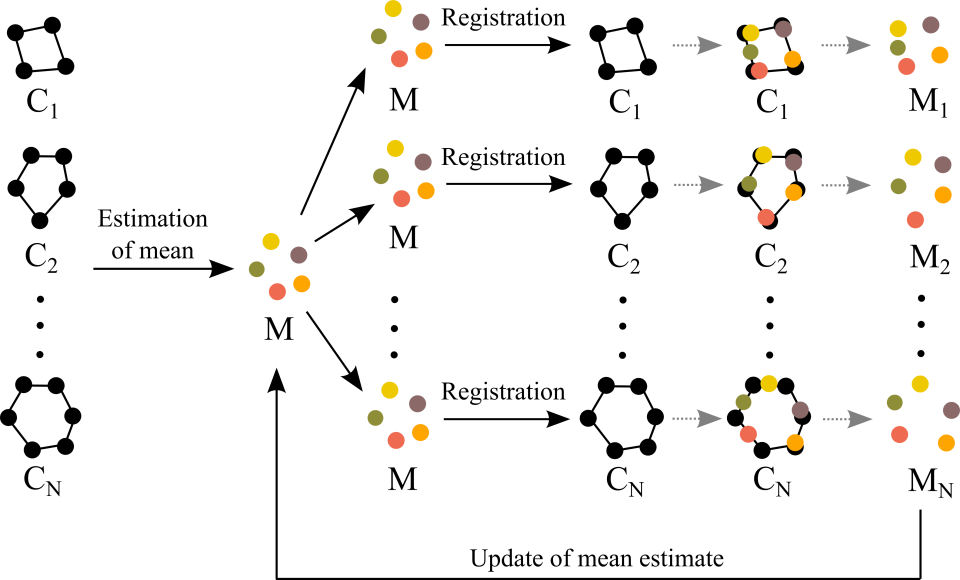


Fig. S1 Mean shape *M* is initially estimated using the information of *N* number of point clouds (*C*_1_, *C*_2,…_, *C_N_*). *N* number of copies of *M* are registered to each point cloud (*C*_1_, *C*_2,…_, *C_N_*). *M_i_* (*i* = 1,…, *N*) represents deformed forms of *M* and corresponding points between clouds *M* and *M_i_* (*i* = 1,…, *N*) have the same colors*.* The new estimate of the mean shape *M* is computed based on the mean of *M_i_* (Tümer et al., 2016)*.*

The registration algorithm mainly consists of the following steps:

1. All point clouds are pre-aligned. During the pre-alignment (i.e. the minimization of differences due to position and scaling of point clouds), each point cloud was translated to the origin of a common coordinate system by averaging *x*, *y*, and *z* coordinates of the points and subtracting them from the original *x*, *y*, and *z* coordinates, respectively. Besides, point clouds are scaled to unit centroid size, which is known as the square root of the sum of squared distances of a set of landmarks from their centroid (Mitteroecker et al., 2013).
2. An initial mean cloud *M* with *n*_m_ points is estimated using the information of the pre-aligned surfaces from which the point clouds *C_i_* (*i* = 1,…, *N*) were obtained (Fig. S1):
   1. Each of the pre-aligned surfaces consisting the points *C_i_* is represented by a signed transform, with the surface as the 0-level.
   2. The signed transforms are averaged and a marching cubes method is applied on the 0-level of it to obtain the surface of the mean shape.
   3. The surface found in Step 2.2. is sampled with *n_m_* points to get the initial mean cloud.
3. The estimated mean cloud *M* is registered to each cloud *C_i_* using the *L*_2_ divergence as a similarity measure between *C_i_* and *M_i_*, which is the deformed form of *M* (Fig. S1).
4. The current estimate of the mean shape *M* is updated based on the mean of all *M_i_* (*i* = 1,…, *N*) (Fig. S1).
5. Steps 3-4 are repeated, if the convergence is not reached. Otherwise, Step 6 is followed.
6. The point clouds *C_i_* (*i* = 1,…, *N*) are transformed to the mean cloud using the correspondence relation between *M_i_* and *M.*

The registration scheme requires a few parameters to be set by the user: the scale parameter *σ* for the mixture of Gaussians, the number of points in the mean cloud *n*_m_, and the regularization term *λ*. The parameters *n*_m_ and *σ* determine the accuracy of the estimated correspondences established during the registration process. The parameter *λ* is used to balance the data misfit and the deformation. The parameters necessary for registering tali were retrieved from (Tümer et al., 2016) and numerical experiments were conducted to determine those required for registering tibiae, fibulae, and calcanei. The numerical experiments were performed in a way described previously (van de Giessen et al., 2012; Tümer et al., 2016).

The important steps followed to determine the parameters *n*_m_ and *σ* were (Tümer et al., 2016):

1. A triangulated surface of a single bone sample was taken and randomly sampled with *n*_m_ points three times to obtain three different point clouds *C_1_*, *C_2_* and *C_3_*.
2. The point clouds *C_1_*, *C_2_* and *C_3_* were registered using the unbiased registration algorithm.
3. The point clouds *C_1_*, *C_2_* and *C_3_* were transformed to the mean cloud using the correspondence established between *M_i_* and *M.*
4. The mean signed point-to-plane distance between all pairs of clouds *C_i_* and *C_j_*, namely the registration accuracy (*E_acc_*) was computed using:

| $E_{acc}= \frac{1}{N(N-1)}\sum_{i=1}^{N} \sum_{j=1\backslash i}^{N} \left\vert\frac{1}{n_{i}}\sum_{k=1}^{n_{i}} \mathbf{u}_{k}\cdot\left( \mathbf{x}_{k}-\mathbf{x}_{k}^{c} \right) \right\vert$ | (1) |
| --- | --- |

where *n_i_* is the number of points in the cloud *C_i_*, **u***_k_* is the normal vector at point **x**_k_ in point cloud *C_i_* and **x**_k_^c^ is the point in cloud *C_j_* closest to **x**_k_.

1. The average point-to-plane distance between all pairs of clouds *C_i_* and *C_j_*, namely the registration precision (*E_prec_*) was computed using:

| $E_{prec}= \frac{1}{N(N-1)}\sum_{i=1}^{N} \sum_{j=1\backslash i}^{N} \frac{1}{n_{i}}\sum_{k=1}^{n_{i}} \left\vert\mathbf{u}_{k}\cdot\left( \mathbf{x}_{k}-\mathbf{x}_{k}^{c} \right) \right\vert$ | (2) |
| --- | --- |

The Steps 1-5 were repeated for all combinations of *σ* ϵ {0.3, 0.6, 1.5, 3, 6, 15}, *n*_m_ ϵ {100, 200, 500, 1000, 2000} and for 15 bone (i.e. tibia, calcaneus, fibula) surfaces. During the experiment, the regularization term *λ* was kept as 10^-9^.

The steps followed to determine the parameter *λ* were (Tümer et al., 2016):

1. Three different bone surfaces were randomly selected and sampled with a fixed number of points (*n*_m_ = 2000) to obtain the point clouds *C_1_*, *C_2_* and *C_3_*.
2. The point clouds *C_1_*, *C_2_* and *C_3_* were registered using the unbiased registration algorithm.
3. The point clouds *C_1_*, *C_2_* and *C_3_* were transformed to the mean cloud using the correspondence established between *M_i_* and *M.*
4. The registration accuracy and precision was computed using Eq. 1 and Eq. 2, respectively.

The Steps 1-4 were repeated for all combinations of *σ* ϵ {0.3, 0.6, 1.5, 3, 6, 15}, *λ* ϵ {10^-8^, 10^-7^, …, 10^-3^} and for 15 random selections of bone (i.e. tibia, calcaneus, fibula) surfaces.

1. **Statistical analyses of ipsi- and contralateral sides as separate groups**

This section covers some important details about the methods mentioned in the section “Statistical analyses ipsi- and contralateral sides as separate groups” of the main manuscript.

- 1. **Dense correspondence established on all aligned bone surfaces of the same type**

To establish a dense correspondence between each pair of aligned bone surfaces of the same type, coordinates of the points and surface normals were used (van de Giessen et al., 2009).

The correspondence between a point **a***_i_* on surface A and a point **b***_ai_* was defined using the minimum Euclidean distance:

| $b_{ai}=\begin{matrix} \arg\min\\ b_{j} \end{matrix}\left\Vert\boldsymbol{a}_{i}-\boldsymbol{b}_{j} \right\Vert$ | (3) |
| --- | --- |

where $\boldsymbol{a}_{i}= \left[ \begin{matrix} \mathbf{p}_{i} \\ \mathbf{n}_{i} \end{matrix} \right]$ and $\boldsymbol{b}_{j}= \left[ \begin{matrix} \mathbf{p}_{j} \\ \mathbf{n}_{j} \end{matrix} \right]$. **p***_i_* and **p***_j_* represent the point coordinates, while **n***_i_* and **n***_j_* stand for the surface normals.

- 1. ***D*-statistic used in group location test**

*D*(*istance*)-statistic used in the group location test was the Euclidean distance between the means of two groups (Claes et al., 2012, 2015). The main steps followed to calculate the *D*-statistic were:

1. Two *N* × *K* matrices **A** and **B** consisting of the coordinates of *m* (i.e. *K = m* × 3) landmarks established on all left (i.e. N = 66) and right (i.e. *N* = 66) side bones of the same type, respectively, were formed:

$$\mathbf{A}= \left( \begin{matrix} {Ax}_{11} & {Ay}_{11} & Az_{11} \\ \vdots& \vdots& \vdots\\ Ax_{N1} & Ay_{N1} & Az_{N1} \end{matrix} \begin{matrix} \ldots\\ \ldots\\ \ldots\end{matrix} \begin{matrix} {Ax}_{1m} & {Ay}_{1m} & Az_{1m} \\ \vdots& \vdots& \vdots\\ Ax_{Nm} & {Ay}_{Nm} & {Az}_{Nm} \end{matrix} \right)$$

$$\mathbf{B}= \left( \begin{matrix} {Bx}_{11} & By_{11} & Bz_{11} \\ \vdots& \vdots& \vdots\\ {Bx}_{N1} & {By}_{N1} & {Bz}_{N1} \end{matrix} \begin{matrix} \ldots\\ \ldots\\ \ldots\end{matrix} \begin{matrix} {Bx}_{1m} & {By}_{1m} & Bz_{1m} \\ \vdots& \vdots& \vdots\\ {Bx}_{Nm} & By_{Nm} & Bz_{Nm} \end{matrix} \right)$$

2. The mean of each column in the matrices **A** and **B**, resulting in $\bar{\mathbf{A}}$ and $\bar{\mathbf{B}}$ is calculated:

$$\bar{\mathbf{A}}\boldsymbol{=} \frac{1}{N}\times\sum_{i=1}^{N} \left( \begin{matrix} {\begin{matrix} {Ax}_{i1} & {Ay}_{i1} & Az_{i1} & \ldots& {Ax}_{im} & {Ay}_{im} & Az \end{matrix}}_{im} \end{matrix} \right)$$

$$\bar{\mathbf{B}}\boldsymbol{=} \frac{1}{N}\times\sum_{i=1}^{N} \left( \begin{matrix} {\begin{matrix} {Bx}_{i1} & By_{i1} & {Bz}_{i1} & \ldots& {Bx}_{im} & By_{im} & Bz \end{matrix}}_{im} \end{matrix} \right)$$

3. The matrix $\bar{\mathbf{B}}$ is subtracted from the matrix $\bar{\mathbf{A}}$ in element-wise manner and the square of each difference is calculated.

$$\bar{\mathbf{A}}\boldsymbol{-}\bar{\mathbf{B}}\boldsymbol{=} \left[ \begin{matrix} \left( \bar{Ax}_{1}-\bar{Bx}_{1} \right)^{2} & \left( \bar{Ay}_{1}-\bar{By}_{1} \right)^{2} & \left( \bar{Az}_{1}-\bar{Bz}_{1} \right)^{2} & \ldots& \left( \bar{Ax}_{m}-\bar{Bx}_{m} \right)^{2} & \left( \bar{Ay}_{m}-\bar{By}_{m} \right)^{2} & \left( \bar{Az}_{m}-\bar{Bz}_{m} \right)^{2} \end{matrix} \right]$$

4. All elements of the matrix obtained in Step 3 was summed and the square root of it was calculated.
   1. ***D*-statistic used in variance-covariance scale test**

*D*-statistic used in the variance-covariance scale test was the absolute difference in the average residual of the two groups (Claes et al., 2012, 2015). The main steps followed to calculate the *D*-statistic were:

1. Steps 1-2 described in the previous section were followed.
2. The matrices $\bar{\mathbf{A}}$ and $\bar{\mathbf{B}}$ were subtracted from the matrices **A** and **B**, respectively. The square of each difference is calculated, summed and the square root of the sum was calculated.

$$\mathbf{A}\boldsymbol{-}\bar{\mathbf{A}}\boldsymbol{=}\mathbf{C}\boldsymbol{=} \left[ \begin{matrix} \sqrt{\left( {Ax}_{11}-\bar{Ax}_{1} \right)^{2}+ \left( {Ay}_{11}-\bar{Ay}_{1} \right)^{2}+ \ldots+\left( {Ay}_{1m}-\bar{Ay}_{m} \right)^{2}+ \left( {Az}_{1m}-\bar{Az}_{m} \right)^{2}} \\ \vdots\\ \vdots\\ \vdots\\ \sqrt{\left( {Ax}_{N1}-\bar{Ax}_{1} \right)^{2}+ \left( {Ay}_{N1}-\bar{Ay}_{1} \right)^{2}+ \ldots+\left( {Ay}_{Nm}-\bar{Ay}_{m} \right)^{2}+ \left( {Az}_{Nm}-\bar{Az}_{m} \right)^{2}} \end{matrix} \right]$$

$$\mathbf{B}\boldsymbol{-}\bar{\mathbf{B}}\boldsymbol{=}\mathbf{D}\boldsymbol{=} \left[ \begin{matrix} \sqrt{\left( {Bx}_{11}-\bar{Bx}_{1} \right)^{2}+ \left( {By}_{11}-\bar{By}_{1} \right)^{2}+ \ldots+\left( {By}_{1m}-\bar{By}_{m} \right)^{2}+ \left( {Bz}_{1m}-\bar{Bz}_{m} \right)^{2}} \\ \vdots\\ \vdots\\ \vdots\\ \sqrt{\left( {Bx}_{N1}-\bar{Bx}_{1} \right)^{2}+ \left( {By}_{N1}-\bar{By}_{1} \right)^{2}+ \ldots+\left( {By}_{Nm}-\bar{By}_{m} \right)^{2}+ \left( {Bz}_{Nm}-\bar{Bz}_{m} \right)^{2}} \end{matrix} \right]$$

3. The mean of the matrices **C** and **D** was calculated, $\bar{\mathbf{C}}$ and $\bar{\mathbf{D}}$.
4. The square root of the squared difference between $\bar{\mathbf{C}}$ and $\bar{\mathbf{D}}$ was calculated.
   1. **Parallel analysis**

Parallel analysis (PA) (Franklin et al., 1995; Ledesma and Valero-Mora, 2007) was performed by generating a random dataset that has an equal dimension with that of the original data (i.e. same sample size and number of variables). Eigenvalues were derived from the random dataset. Generation of the random dataset and extraction of eigenvalues were repeated 50 times. The 95^th^ of the distribution of the replicated eigenvalues (i.e. 95^th^ percentile) for each component was determined. A principal component was kept if the associated eigenvalue (i.e. the one derived from the original data) was bigger than the calculated 95^th^ percentile.

1. **Statistical analyses of ipsi- and contralateral sides pooled into one group**

This section covers an explanation for the term “shape parameters” mentioned in the section “Statistical analyses ipsi- and contralateral sides pooled into one group” of the main manuscript.

- 1. **Shape parameters**

A new or existing shape (**x**) can be represented using the mean shape $\bar{\mathbf{x}}$ and a weighted summation of shape variance directions (i.e. PCs, eigenvectors or modes of shape variation) (**Ф**), as (Sarkalkan et al., 2014):

$$\mathbf{x}= \bar{\mathbf{x}}+ \sum_{s=1}^{c} b_{s}\boldsymbol{\Phi}_{s}$$

in which, *b*_s_ values (i.e. weights or shape parameters) express the contributions of the first c modes of shape variation to the mean bone shape.

**REFERENCES**

**Claes P, Reijniers J, Shriver MD**, et al. (2015) An investigation of matching symmetry in the human pinnae with possible implications for 3D ear recognition and sound localization. *J Anat* **226**, 60–72.

**Claes P, Walters M, Shriver MD, et al.** (2012) Sexual dimorphism in multiple aspects of 3D facial symmetry and asymmetry defined by spatially dense geometric morphometrics. *J Anat* **221**, 97–114.

**Franklin SB, Gibson DJ, Robertson PA, et al.** (1995) Parallel analysis: a method for determining significant principal components. *Source J Veg Sci J Veg Sci* **6**, 99–106.

**Giessen M van de, Smitsman N, Strackee SD, et al.** (2009) A statistical description of the articulating ulna surface for prosthesis design. *Proc - 2009 IEEE Int Symp Biomed Imaging From Nano to Macro*, 678–681.

**Giessen M van de, Vos FM, Grimbergen CA, et al.** (2012) An efficient and robust algorithm for parallel groupwise registration of bone surfaces. *Med Image Comput Comput Assist Interv* **15**, 164–71.

**Ledesma RD, Valero-Mora P** (2007) Determining the number of factors to retain in EFA: an easy-to-use computer program for carrying out parallel analysis. *Res Eval* **12**, 1–11.

**Mitteroecker P, Gunz P, Windhager S,** et al. (2013) A brief review of shape, form, and allometry in geometric morphometrics, with applications to human facial morphology. *Hystrix* **24**, 59–66.

**Sarkalkan N, Weinans H, Zadpoor AA** (2014) Statistical shape and appearance models of bones. *Bone* **60**, 129–140.

**Tümer N, Blankevoort L, Giessen M Van De, et al.** (2016) Bone shape difference between control and osteochondral defect groups of the ankle joint. *Osteoarthr Cartil* **24**, 2108–2115.
